# Supplementary material for: Novel data-driven subtypes and stages of brain atrophy in the ALS-FTD spectrum
Source: Res Sq. 2023 Aug 10:rs.3.rs-3183113. Preprint. [Version 1] doi: 10.21203/rs.3.rs-3183113/v1 (PMC10441467; doi:10.21203/rs.3.rs-3183113/v1)
Supplement: Supplement 1 [file NIHPPrs3183113v1-supplement-1.pdf]

Supplementary Files

This is a list of supplementary files associated with this preprint. Click to download.

- SupplementaryMaterial.docx
